# Research on social and economic factors influencing regional mortality patterns in China

**DOI:** 10.1038/s41598-024-61262-5

**Published:** 2024-05-09

**Authors:** Tiantian Li, Shuyin Zhang, Handong Li

**Affiliations:** School of Systems Science, Beijing Normal University, Beijing, China

**Keywords:** Mortality patterns, Regional disparities, Determining factors, Functional regression, Health care, Risk factors

## Abstract

Regional population mortality correlates with regional socioeconomic development. This study aimed to identify the key socioeconomic factors influencing mortality patterns in Chinese provinces. Using data from the Seventh Population Census, we analyzed mortality patterns by gender and urban‒rural division in 31 provinces. Using a functional regression model, we assessed the influence of fourteen indicators on mortality patterns. Main findings: (1) China shows notable gender and urban‒rural mortality variations across age groups. Males generally have higher mortality than females, and rural areas experience elevated mortality rates compared to urban areas. Mortality in individuals younger than 40 years is influenced mainly by urban‒rural factors, with gender becoming more noticeable in the 40–84 age group. (2) The substantial marginal impact of socioeconomic factors on mortality patterns generally becomes evident after the age of 45, with less pronounced differences in their impact on early-life mortality patterns. (3) Various factors have age-specific impacts on mortality. Education has a negative effect on mortality in individuals aged 0–29, extending to those aged 30–59 and diminishing in older age groups. Urbanization positively influences the probability of death in individuals aged 45–54 years, while the impact of traffic accidents increases with age. Among elderly people, the effect of socioeconomic variables is smaller, highlighting the intricate and heterogeneous nature of these influences and acknowledging certain limitations.

## Introduction

The distribution of mortality probability by age defines the mortality pattern of a population. Regional population mortality patterns portray the probability distribution of deaths across various age groups in a specific geographic area during a given period. Typically, population censuses categorize individuals into age groups such as 0 years, 1–4 years, …, 95–99 years, and 100 years and above, for a total of 22 groups. Mortality statistics for each age group are collected, and age-specific mortality rates are converted into probabilities to derive population mortality patterns. Population mortality patterns, influenced by biomedical, demographic, socioeconomic, and environmental factors, are crucial indicators reflecting demographic changes. Among these factors, socioeconomic factors play a significant role in shaping regional population mortality patterns and constitute a key focus of demographic studies.

China, characterized by a vast population and distinct urban‒rural dual social structure with extreme regional economic disparities, offers different opportunities and choices for accessing social resources and improving quality of life. This, in turn, impacts mortality patterns in regional populations. This paper aims to answer key questions: What are the primary socioeconomic factors influencing disparities in regional mortality patterns in China? How do these factors affect the distribution of age-specific mortality probabilities in regional populations, and to what extent? Addressing these questions is the primary focus of this research.

The results of this paper provide an important decision-making basis for the government to formulate regional development policies, rationally allocate public medical and health resources, promote sustainable regional social and economic development, and improve regional population health inequality.

The remaining sections of this paper are structured as follows: Section “Literature review” presents a literature review, section “Research method and variable selection” details the research methods and variable selection, section “Empirical analysis” provides the empirical analysis, and the final section contains the conclusion.

## Literature review

The exploration of population mortality and its determinants is a key area in population research, yielding extensive findings. From a socioeconomic perspective, research on regional mortality involves understanding and contrasting patterns, examining socioeconomic influences, and assessing methodological approaches.

### Characteristics and comparisons of regional mortality patterns

To explore the temporal and spatial relationships of regional mortality patterns, numerous scholars have conducted comprehensive and extensive research on mortality patterns in various countries and regions, including the United States, Germany, New Zealand, Russia, South Korea, and China, among others, yielding substantial results.

Ivanova & Zhong analyzed regional disparities in male mortality rate changes in Russia, revealing significant stratification^1^. Kibele et al. studied German regional mortality rates from 1910 to 2009, noting substantial changes over the century^2^. Van Raalte et al. used Germany as an example and reported that significant regional economic inequality does not necessarily parallel disparities in mortality rates^3^. Spencer et al. decomposed differences in mortality rates between US urban and rural counties, attributing the widening gap to changes in how characteristics influence mortality^4^.

Many studies have also focused on changes in mortality rates and their distribution in China. Cao et al. reported varying child mortality rates across 81 cities and counties, with higher rates for males overall but a reversal in impoverished areas^5^. Nie and Song reported an uneven regional distribution of infant mortality rates, increasing from coastal to inland and then to remote regions^6^. Li compared mortality levels in North and East China^7^. Zhou, using cluster analysis, explored regional variations, finding influences of both geographical location and economic conditions^8^. Chen et al. used spatial analysis to characterize the spatiotemporal patterns of urban population mortality rates in China, revealing distinct spatial heterogeneity and correlations^9^.

### Factors influencing mortality patterns research

Mackenbach et al. conducted a regression-based analysis comparing inequality indices in mortality and self-rated health across 22 European countries, revealing higher mortality rates and poorer self-rated health in socioeconomically disadvantaged groups^10^. Kibele et al. found that factors contributing to spatial variations in mortality in Germany changed significantly over time, with a strengthened connection between regional socioeconomic conditions and mortality^2^. Mackenbach's review of theories explaining social inequality suggested that health inequality plays a role in exacerbating social inequality^11^. Lutz and Kebede's study across 174 countries from 1970 to 2010 found education to be a better predictor, linking improved education to increased income and better health outcomes^12^. Gutin and Hummer’s review highlighted the association between social status and health, where high socioeconomic status individuals may extend their lives beyond biological expectations, while disadvantages due to social status could lead to premature mortality for lower-status individuals^13^.

Several studies have investigated the intricacies of factors affecting mortality rates in China. Zhao's macrolevel exploration identified socioeconomic factors impacting post-1949 mortality rates^14^. Nie & Song empirically analyzed the factors that influenced infant mortality rates between 1996 and 2002^0^. Luo & Xie uncovered contextual determinants for the influence of socioeconomic indicators on elderly mortality, highlighting the nuanced sociopolitical landscape of China^15^. Li's work revealed the significant impact of healthcare resource investment on regional disparities in elderly health^16^. Chen et al. conducted a comprehensive investigation into various factors shaping population mortality, spanning from wastewater pollution to healthcare improvements^17^. Fan’s latent socioeconomic status variable stressed the pivotal role of higher socioeconomic status in mitigating mortality risk among Chinese individuals aged 65 and above^18^. Chen et al.’s analysis of PM2.5 pollution demonstrated a positive correlation with mortality rates, coupled with spatial spillover effects^9^. Ying & Li highlighted the prominent role of population aging, industrial emissions, climatic conditions, and per capita GDP in shaping mortality rates^19^.

### Research methods for regional mortality patterns

In the study of mortality patterns and the influencing factors, scholars often use statistical regression models and similar methodologies. For instance, Lutz & Kebede employed multivariate statistical analysis to explore the impacts of education and income on global mortality patterns^12^. The Cox proportional hazards model is a common choice, as seen in the work of Luo & Xie, who used it to study the mechanisms through which socioeconomic factors influence mortality rates in China^15^. Fan also utilized the Cox proportional hazards regression model to compute hazard ratios and conducted subgroup analyses based on various factors^18^. Other techniques, such as multiple regression analysis^9^, global spatial autocorrelation models, local spatial autocorrelation models, and spatial regression analysis methods^17^, are employed to investigate various aspects of regional mortality patterns.

In summary, current research on regional mortality disparities in China faces several limitations. First, prior studies often attribute differences in mortality rates to a limited set of socioeconomic factors, and a comprehensive analysis of the overarching socioeconomic forces is lacking. Second, there is a predominant focus on socioeconomic impacts within specific age groups, with insufficient analyses covering the entire mortality pattern. Third, most studies use classical statistical regression analyses, while this paper adopts more recent functional regression models, a method still underutilized in population studies.

## Research method and variable selection

In this study, a mortality pattern was employed as a measure of regional population mortality levels. The mortality pattern represents the distribution of age-specific mortality probabilities and can be considered a function of population age. We utilized the function-on-scalar functional regression model for our analysis, drawing inspiration from the foundational principles outlined in the works of J.O. Ramsay and B.W. Silverman^20,21^ and relevant theoretical literature^22–29^. The basic principles of the functional regression model are outlined as follows.

### Representation of functional data

The subject of functional data analysis deals with a set of smooth curves, denoted as $\left\{{y}_{n}^{0}\left(t\right), t\in \left[{T}_{0},{T}_{1}\right], 1\le n\le N\right\}$, comprising N such curves. For a given sample curve, we can only observe a finite number of points, i.e., $\{{y}_{n,j}^{0}\in R, 1\le n\le N, 1\le j\le {J}_{n}\}$, with each curve having ${J}_{n}$ observed points. Functional data possess infinite dimensional characteristics, and for the sake of data modeling and statistical inference, dimension reduction techniques are often applied. One common approach is to expand the functional data using basis functions. Given the known discrete points, fitting with basis functions allows the representation of the original functional data as a linear combination of these basis functions. Let $\{{\phi }_{k}, 1\le k\le K\}$ be the basis functions, then the fitted function can be expressed as follows:

1 $${y}_{n}(t)\approx {\sum }_{k=1}^{K}{c}_{n,k}{\phi }_{k}\left(t\right),1\le n\le N$$

where ${c}_{n,k}$ represents the coefficient for each basis function ${\phi }_{k}\left(t\right)$, and the original data can be expressed as follows:

2 $${y}_{n,j}^{0}={y}_{n}\left({t}_{j,n}\right)+{\varepsilon }_{n,j}, 1\le n\le N, {t}_{j,n}\in \left[{T}_{0},{T}_{1}\right], 1\le j\le {J}_{n}$$

where ${\varepsilon }_{n,j}$ represents the error terms, which are independently and identically distributed with a mean of zero and a variance of ${\sigma }^{2}$.

The basis functions can be specified as periodic Fourier bases, nonperiodic B-spline bases, or wavelet bases. Alternatively, data-driven functional principal component bases can be selected, but this necessitates performing principal component analysis (PCA) on the functional data beforehand.

If the original data are affected by a significant level of noise, the expansion of the basis functions becomes increasingly distorted as the number of basis functions increases. Having a relatively small number of basis functions, on the other hand, will also limit the shape of their linear combinations. Therefore, we employ a roughness penalty method to smooth the functional data:

3 $${PSS}_{\lambda }\left({c}_{n,k}\right)={\sum }_{j=1}^{{J}_{n}}{\left[{y}_{n,j}^{0}-{y}_{n}\left({t}_{j,n}\right)\right]}^{2}+\lambda {\int }_{{T}_{0}}^{{T}_{1}}[{\mathcal{L}{y}_{n}\left(t\right)]}^{2}dt$$

At this point, we need to find the coefficients ${c}_{n,k}$ to minimize the penalty square sum, where $\lambda \in {R}_{+}$ is the smoothing parameter, and $\mathcal{L}$ is a linear differential operator. To strike a balance between overfitting and oversmoothing by adjusting $\lambda$, we utilized the generalized cross-validation (GCV) method.

### Functional regression model

Functional regression analysis encompasses various models, such as scalar-to-function regression, function-to-scalar regression, function-to-function regression, mixed-variable regression, nonlinear regression of scalars to functions, and functional generalized linear models. Given the specific problem under investigation, this paper employs a function-to-scalar regression analysis model, expressed as follows:

4 $${y}_{n}\left(t\right)={x}_{n1}{\beta }_{1}\left(t\right)+{x}_{n2}{\beta }_{2}\left(t\right)+\dots +{x}_{nq}{\beta }_{q}\left(t\right)+{\varepsilon }_{n}\left(t\right) , 1\le n\le N$$

The function variable ${y}_{n}\left(t\right), 1\le n\le N$, observed $N$ times, is an N-dimensional vector. It needs to be generated using the observed values $\{{y}_{n,j}^{0}\in R, 1\le n\le N, 1\le j\le {J}_{n}\}$ from the initial data. ${x}_{ni},1\le n\le N, 1\le i\le q$ is a set of $q$ covariate scalars. ${\beta }_{i}\left(t\right), 1\le i\le q$ represents q functional regression parameters, also known as effect functions, which can be expressed using the basis function ${\varphi }_{1},{\varphi }_{2},\dots ,{\varphi }_{K}$ as ${\beta }_{i}\left(t\right)={\sum }_{k=1}^{K}{b}_{ik}{\varphi }_{k}(t), 1\le i\le q$, and ${b}_{ik}, 1\le i\le q, 1\le k\le K$ represents the coefficient for each basis function ${\phi }_{k}\left(t\right)$. ${\varepsilon }_{n}\left(t\right), 1\le n\le N$ represents the error term function.

Assuming that the observation points are $\{{t}_{j}:1\le j\le J\}$, the model can be represented in matrix form as:

5 $$\mathbf{Y}=\mathbf{X}\mathbf{B}{\varvec{\Phi}}+{\varvec{\upvarepsilon}}$$

Here,

$$\mathbf{Y}={({Y}_{n}({t}_{j}))}_{1\le n\le N}^{1\le j\le J} , \mathbf{X}={({x}_{ni})}_{1\le n\le N}^{1\le i\le q} , \mathbf{B}={({b}_{ik})}_{1\le i\le q}^{1\le k\le K} , {\varvec{\Phi}}={({\varphi }_{k}({t}_{j}))}_{1\le k\le K}^{1\le j\le J} , {\varvec{\upvarepsilon}}={({\varepsilon }_{n}({t}_{j}))}_{1\le n\le N}^{\begin{array}{c}1\le j\le J\end{array}}.$$

As a result, the penalized least squares used for the regression can be defined as:

6 $${P}_{\lambda }\left(B\right)={\sum }_{n=1}^{N}{\sum }_{j=1}^{J}{[{Y}_{n}\left({t}_{j}\right)-{(\mathbf{X}\mathbf{B}{\varvec{\Phi}})}_{nj}]}^{2}+{\sum }_{i=1}^{q}{\lambda }_{i}\int {[\mathcal{L}{\beta }_{i}(t)]}^{2}dt$$

Similarly, we need to find the coefficients $\mathbf{B}$ to minimize the sum of squares with the penalty mentioned above. In this paper, we assume that a single smoothing parameter ${\lambda }_{i}, 1\le i\le q$ imparts a uniform level of smoothness to each component, and we employ the smoothing parameter cross-validation (CV) method for selection.

The advantage of using functional data regression analysis lies in its consideration of the holistic nature of the functional dependent variable. Our study focuses on the overall mortality pattern, representing a complete function curve. In comparison to traditional multivariate regression methods, functional regression analysis avoids separate regressions for different age groups and instead comprehensively considers the overall variation. Multivariate regression methods often perform local fitting, providing accurate results in specific age ranges but lacking a comprehensive study of the overall mortality pattern and the identification of factors influencing the entire pattern. In supplementary information II, we present a comparative analysis between the results of multivariate regression methods and our approach, demonstrating that functional data regression, in contrast to traditional methods, more comprehensively reveals the characteristics of the mortality pattern. This global analysis contributes to a deeper understanding of the overall patterns in population mortality.

### Functional hypothesis testing

In the empirical section, two hypothesis tests were employed: the functional F test and the pointwise t test. The pointwise t test is analogous to the traditional t test, with the initial step involving discretization of functional data as needed, followed by steps identical to those in the traditional t test. Further details are not provided here. The following section introduces the functional F test method derived from functional regression analysis^27,30^.

Without loss of generality, we assume the original model to be

7 $$Y(t)={X}_{1}{\beta }_{1}(t)+{\dots +X}_{p}{\beta }_{p}(t)+\epsilon (t)$$

Write the alternative hypothesis (Model 1) as follows:

8 $${H}_{1}:Y(t)={X}_{1}{\beta }_{1}(t)+{\dots +X}_{p}{\beta }_{p}(t)+\epsilon (t)$$

Write the null hypothesis (Model 0i) as:

9 $${H}_{0i}:{ \beta }_{i}\left(t\right)=0, i=1,\dots ,p$$

The functional F test statistic is defined as follows:

10 $${\mathcal{F}}_{i}=\frac{rs{s}_{0i}-rs{s}_{1}}{rs{s}_{1}/(n-p)}$$

Here, $ss=\sum_{n=1}^{N}\int {({y}_{n}(t)-{\widehat{y}}_{n}(t))}^{2}dt$, ${y}_{n}(t)$ represents the initial functional curve, and ${\widehat{y}}_{n}(t)$ represents the regression-predicted functional curve.

We reject the null hypothesis if ${\mathcal{F}}_{i}>{F}^{(1-\alpha )}({f}_{1},{f}_{2})$, where ${f}_{1}=[{\text{trace}}(E{)}^{2}/{\text{trace}}({E}^{2})], {f}_{2}=[(n-p){\text{trace}}(E{)}^{2}/{\text{trace}}({E}^{2})],$ and $E$ is the empirical covariance matrix of the error process obtained from the alternative hypothesis.

### Data processing and variable selection

The data for this study were derived from the 2020 *China Population Census Yearbook* published by the National Bureau of Statistics of China, the 2021 *China Health Statistics Yearbook*, and annual provincial-level data from the *National Data Website*. These data are given in Online Resource 1. The dataset covers all 31 provinces in mainland China. The dependent variable data consist of mortality probabilities by age, gender, and the urban‒rural classification for each of the 31 provincial administrative regions. The independent variables encompass gender, the urban‒rural classification, and various socioeconomic variables. Variable selection was carried out through the following steps:

First, we utilized provincial mortality rate data from the Seventh National Population Census as the basis for estimating mortality probabilities for 22 age groups. Mortality probabilities were calculated for both gender and urban‒rural subgroups, serving as measures of mortality levels for the 31 provinces.

The original dependent variable data were sex and the urban/rural age-specific mortality probabilities for 31 provincial districts. The age-specific mortality probabilities are the probability that the number of people who have reached a certain age will die before reaching another specific age, usually calculated from the known mortality rate^31^. The age-specific mortality rate data were obtained from the 7th Chinese census, and the formula was

11 $${m}_{x}=\frac{{D}_{x}}{{P}_{x}}$$

where ${m}_{x}$ represents the mortality rate at the age of $x$, ${D}_{x}$ represents the number of deaths in the age group of $x$, and ${P}_{x}$ represents the population in the age group of $x$.

If the age group distance is $n$, the age-specific mortality probabilities can be expressed as

12 $${q}_{x}=\frac{{2}^{*}{n}^{*}{m}_{x}}{2+{n}^{*}{m}_{x}} (n\mathrm{ years interval})$$

When $$x=0$$:

13 $${q}_{0}=\frac{{m}_{0}}{1+(1-r){m}_{0}}$$

In the last age group, the age-specific mortality probabilities are equal to 1.

Based on the Seventh National Population Census data, we obtained mortality probabilities for 22 age groups: 0 years, 1–4 years, 5–9 years, 10–14 years, 15–19 years, 20–24 years, 25–29 years, 30–34 years, 35–39 years, 40–44 years, 45–49 years, 50–54 years, 55–59 years, 60–64 years, 65–69 years, 70–74 years, 75–79 years, 80–84 years, 85–89 years, 90–94 years, 95–99 years and 100 years and above. Since the mortality probability for individuals aged 100 years and above reaches a limiting value of 1 and the data for the 95–99 years age group exhibit substantial variations across provinces, these two age groups were excluded from our analysis.

Second, the impact of socioeconomic factors on population mortality levels is multifaceted. Previous research has established a strong correlation between the level of socioeconomic development, quality of life, medical care, and technological advancements in a region. Additionally, higher levels of education have been shown to effectively reduce mortality probabilities. Drawing from prior studies on the socioeconomic factors influencing mortality patterns and considering the comprehensiveness and availability of data, we selected 22 indicators. These indicators were subjected to functional stepwise regression analysis, and variables with poor regression performance and nonsignificant regression coefficients were removed. Based on previous experience, we initially selected seven indicators, including Per Capita Gross Domestic Product (PGDP), the urbanization rate, education, gender, and the urban‒rural status, to construct the foundational functional regression model. The model's coefficient of determination (R^2^) was relatively low. To enhance the model's performance, we gradually introduced different factor indicators and conducted variable selection based on p values from pointwise t tests.

During the stepwise introduction and screening of variables, we used pointwise t test *p* values as the criterion, considering the initial twenty age segments as data points for testing the regression coefficients of each factor. If all values in the p value vector were greater than 0.05, we removed that factor and continued to introduce new factors, repeating the process. Simultaneously, we removed certain independent variables, examined changes in the overall model's pointwise t test significance, and gradually selected statistically significant variables affecting the target variable, resulting in a relatively concise and effective model.

In the final model, although the variables health technicians and insured employees did not pass the pointwise significance test at the 0.05 level, they were important medical human resource indicators and social security variables, and therefore, were retained. Additionally, considering the overall p values of all independent variables, their inclusion strengthened the pointwise significance of all variables. The model also included two resource-related medical indicators (hospital beds) and social security variables (insured residents) corresponding to them. Possible collinearity issues may have resulted in lower significance for the former two variables. The retention of these variables also facilitated a comparative analysis of their marginal effects with the corresponding variables.

Ultimately, we selected the gender indicator and thirteen socioeconomic indicators that influence regional mortality patterns, as shown in Table 1, and categorized them into three groups: demographic attributes, economic attributes, and social attributes.

**Table 1:** Attributes and definitions.

Category	Attribute Name	Attribute Definition
Demographic Attributes	Mortality Probabilities	Age-specific mortality probabilities. An important indicator for assessing regional population mortality patterns
	Gender	A control variable: equal to 1 for males and 0 for females
Economic Attributes	PGDP	Per capita GDP. Measures the wealth or ability to withstand social risks possessed by the population
	Tertiary Sector	Proportion of the tertiary sector (%). Reflects the level of transformation and development in the regional economic structure
Social attributes	Urban‒Rural	A control variable for urban and rural classification, with a value of 1 indicating urban and 0 indicating rural
	Urbanization	Urbanization rate (%). A measure of urbanization level and the urbanization process
	Income	Per capita disposable income of urban and rural residents (CNY). Measuring the income level of the population, thereby reflecting residents' living standards and well-being^32^
	Consumption	Per capita consumption expenditure of urban and rural residents (CNY). An important indicator reflecting the standard of living and quality of life of residents
	Hospital Beds	Number of hospital beds per 10,000 people. An important physical resource indicator for evaluating the supply capacity of medical resources
	Health Technicians	Number of health technicians per 10,000 people. An important human resource indicator for evaluating the supply capacity of medical resources
	Fatality Rate	Mortality rate of medical and health institutions (%). An indicator reflecting the state of the regional healthcare system and its level of development
	Insured Residents	Proportion of urban and rural residents covered by basic medical insurance (%). An indicator reflecting the coverage of social security for non-working populations
	Insured Employees	Proportion of employees covered by basic medical insurance (%). An indicator reflecting the coverage of social security for working populations
	Education	Average years of education for the population aged 15 and above (Years). A crucial indicator for measuring the level of education and human capital development
	Traffic Accidents	Total number of traffic accidents. An indicator measuring public safety levels and the risk of population mortality

Source: China Population Census Yearbook, China Health Statistics Yearbook and National Data Website, 2020–2021.

Demographic attribute indicators include age-specific mortality probabilities and gender variables. Economic attributes include PGDP and tertiary sector. Social attributes encompass a variety of indicators. The urban‒rural indicator is a binary control variable with a value of 0 or 1. The urbanization rate serves as an indicator of the urban‒rural development level, while income and consumption represent indicators of residents' living standards. Hospital beds are indicators of physical resource capacity in healthcare development, and health technicians serve as indicators of human resource capacity in healthcare development. The fatality rate is also used as an indicator of the level of healthcare development. Healthcare security level indicators include insured residents and insured employees. These two insurance indicators were calculated based on the number of people covered by basic medical insurance and the total number of permanent residents, as reported in the *National Statistical Yearbook*. Education served as an indicator of educational attainment. Traffic accidents are used as indicators of public safety levels. To standardize the different scales of the data for comparison, we normalized the covariate indicators using Z score transformation based on the mean and standard deviation of the original indicator data.

## Empirical analysis

**Figure 1:**
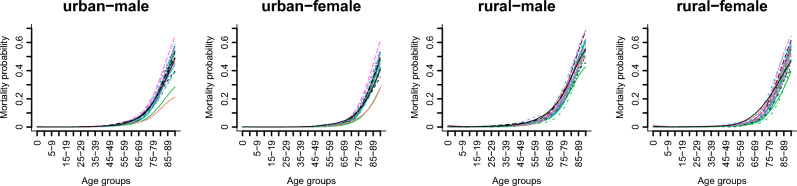
The mortality pattern curves for the 31 provincial regions.

Using the outlined methods and weighted least squares fits, mortality pattern curves for China's provincial regions in 2020 were derived (Fig. 1) and categorized by gender and urban‒rural classification.

Key observations from Fig. 1 include the following: (1) Mortality patterns exhibit a "J"-shaped distribution, with higher probabilities in infants and a gradual increase after age 40, increasing significantly after 70. (2) Females in both urban and rural populations show lower mortality probabilities across age groups than males. (3) Urban populations generally have lower mortality probabilities than do their rural counterparts of the same gender. (4) In urban areas, female mortality increases at approximately age 50, while mortality increases for urban males and rural females at approximately age 45, and rural males show a substantial increase in mortality at approximately age 40. (5) Substantial provincial variation exists, with divergence in mortality patterns for rural males starting at approximately age 35, those for urban males and females starting at approximately age 50, and rural females exhibiting increasing divergence at approximately age 45. Notably, considerable variations exist in mortality probabilities for the elderly population (those over age 60) among provinces.

These findings not only lay the groundwork for identifying the factors influencing mortality patterns regionally but also offer important insights into the mechanisms through which these driving factors affect mortality probabilities across different age groups.

### Overall regression results of the model

We established the functional regression model as follows:

14 $${y}_{i}\left(t\right)={\beta }_{0}\left(t\right)+{GD}_{i}{\beta }_{1}\left(t\right)+{GDP}_{i}{\beta }_{2}\left(t\right)+{TS}_{i}{\beta }_{3}\left(t\right)+{UR}_{i}{\beta }_{4}\left(t\right)+{URR}_{i}{\beta }_{5}\left(t\right)+{PCI}_{i}{\beta }_{6}\left(t\right)+{PCC}_{i}{\beta }_{7}\left(t\right)+{HB}_{i}{\beta }_{8}\left(t\right)+{HT}_{i}{\beta }_{9}\left(t\right)+{FR}_{i}{\beta }_{10}\left(t\right)+{IR}_{i}{\beta }_{11}\left(t\right)+{IE}_{i}{\beta }_{12}\left(t\right)+{EDU}_{i}{\beta }_{13}\left(t\right)+{TA}_{i}{\beta }_{14}\left(t\right)+{\varepsilon }_{i}\left(t\right) ,i=1,...,124$$

**Figure 2:**
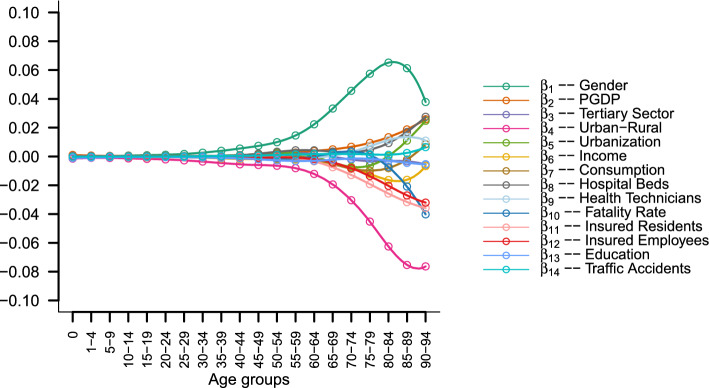
Functional regression coefficient results.

Here, ${y}_{i}\left(t\right)$ represents the smooth curves of mortality probabilities for the four mortality patterns across the 31 provincial regions, totaling 124 curves. ${\beta }_{0}\left(t\right)$ is the functional constant term, ${\varepsilon }_{i}\left(t\right)$ is the functional random error term, and ${\beta }_{1}\left(t\right) \sim {\beta }_{14}\left(t\right)$ are the functional regression coefficients for various variables. The meanings of the other symbols are detailed in Table 2. Figure 2 shows the regression coefficient curves for 14 explanatory variables.

**Table 2:** Descriptive statistics of attributes.

Attribute Name	Symbol	Mean (Standard Deviation)
Gender	GD	
PGDP	GDP	70,786.58(31,325.74)
Tertiary Sector	TS	53.89 (7.57)
Urban–Rural	UR	
Urbanization	URR	62.46(13.47)
Urban Income	PCI	42,252.67(11,129.24)
Rural Income	PCI	17,814.39 (5833.54)
Urban Consumption	PCC	26,080.76(6043.74)
Rural Consumption	PCC	13,848.59(3299.88)
Hospital Beds	HB	64.68(8.72)
Health Technicians	HT	77.61(11.43)
Fatality Rate	FR	0.49 (0.39)
Insured Residents	IR	71.00 (20.55)
Insured Employees	IE	24.32 (14.77)
Education	EDU	9.88(0.99)
Traffic Accidents	TA	7892.71(6096.92)

Urban income and rural income are collectively referred to as 'income' in the analysis, and urban consumption and rural consumption are collectively referred to as 'consumption'.

Examining Fig. 2’s 14 regression coefficient curves reveals that the influence of covariates on regional mortality patterns intensifies notably after age 45. Both demographic and socioeconomic factors show a significant increase in their impact on mortality probabilities, as evidenced by the substantial increase in the absolute values of the regression coefficients. Among the 14 coefficients, gender and urban‒rural status had the greatest impact on overall mortality probabilities. The gender coefficient consistently holds a positive value, indicating higher male mortality probabilities across different age groups, all else being equal. Conversely, the urban‒rural coefficient remains consistently negative, signifying lower mortality probabilities in urban areas than in rural areas across all age groups, attributable to various factors.

**Figure 3:**
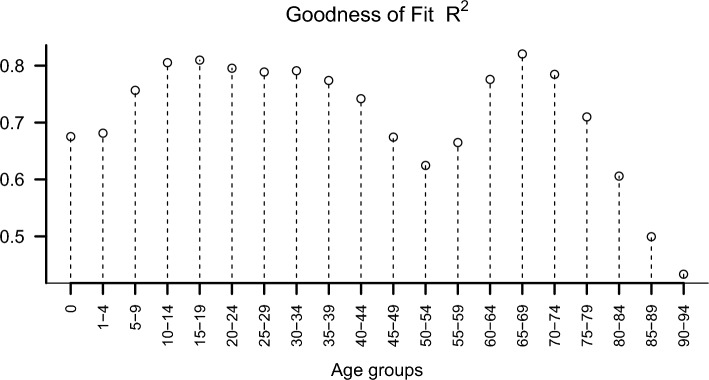
Goodness-of-fit values for each age group.

Figure 3 shows the goodness of fit of the functional regression model by age group.

The model's goodness of fit, which surpassed 60%, notably exceeded 80% for the 10–14 years, 15–19 years, and 65–69 years age groups. In the nine other age groups (5–9 years, 20–24 years, 25–29 years, 30–34 years, 35–39 years, 40–44 years, 60–64 years, 70–74 years, and 75–79 years), the goodness of fit exceeded 70%. This indicates that the 14 selected demographic and socioeconomic indicators effectively explain the mortality probabilities for these age groups. However, the model's fit decreased notably for the older age groups, especially those older than 85 years. This implies the necessity of additional factors to elucidate mortality probabilities for the last two age groups. The lower coefficient of determination ${R}^{2}$ for the last two age groups, particularly for the 90–94 years age group, suggests the influence of unconsidered factors, such as biological and environmental variables, which will be incorporated in future research.

In Table 3, we use the functional F test to test the significance of each coefficient. The variables gender and urban‒rural attributes are significant at the 0.1% level, which is consistent with the analysis above. The remaining two significant functions are hospital beds and fatality rate. In this way, we selected four indicators as global influencing variables. The purpose of testing the functional regression coefficients is to determine whether each regression coefficient function is significantly different from zero. The mortality probabilities are close to zero in the initial age groups (1–39 years), leading to very large and nonsignificant *p* values for the regression coefficients, as these coefficients were already close to zero. However, the lack of significance of the overall functional regression coefficients does not imply that they are not significant in every age group. Subsequently, we conducted pointwise t tests to observe the significance of each functional coefficient in different age groups.

**Table 3:** The *p* values from the functional F tests for the functional regression coefficients.

Attribute category	Functional regression coefficients	The *p*-value from the functional F-test
Demographic attributes	${\beta }_{1}\left(t\right)$– Gender	9.041112e-29***
Economic attributes	${\beta }_{2}\left(t\right)$– PGDP	0.2427897
	${\beta }_{3}\left(t\right)$– Tertiary Sector	0.958046
Social attributes	${\beta }_{4}\left(t\right)$– Urban–Rural	4.689741e-32***
	${\beta }_{5}\left(t\right)$– Urbanization	0.1561508
	${\beta }_{6}\left(t\right)$– Income	0.6637036
	${\beta }_{7}\left(t\right)$– Consumption	0.8381877
	${\beta }_{8}\left(t\right)$– Hospital Beds	0.0004727471***
	${\beta }_{9}\left(t\right)$– Health Technicians	0.1635833
	${\beta }_{10}\left(t\right)$– Fatality Rate	0.0002020485***
	${\beta }_{11}\left(t\right)$– Insured Residents	0.08622965
	${\beta }_{12}\left(t\right)$– Insured Employees	0.6487598
	${\beta }_{13}\left(t\right)$– Education	0.9240515
	${\beta }_{14}\left(t\right)$– Traffic Accidents	0.7131577

One asterisk (*) indicates significance at the 5% level, i.e., *p* < 0.05; two asterisks (**) indicate significance at the 1% level, i.e., *p* < 0.01; three asterisks (***) indicate significance at the 0.1% level, i.e., *p* < 0.001.

### *Analysis of the age-specific effects of the variables gender, urban*‒*rural, hospital beds, and fatality rate*

Table 4 shows the regression coefficient values and significance levels of the variables gender, urban**‒**rural, hospital beds and fatality rate.

**Table 4:** Coefficients and significance of the variables gender, urban‒rural, hospital beds, and fatality rate.

Age groups	${\beta }_{1}\left(t\right)$	${\beta }_{4}\left(t\right)$	${\beta }_{8}\left(t\right)$	${\beta }_{10}\left(t\right)$
0	4.68E-04*	−1.54E-03***	−2.57E-05	−4.82E-05
1–4	1.43E-04	−9.83E-04***	7.93E-05	−1.26E-04
5–9	2.77E-04***	−1.02E-03***	8.99E-05	−1.15E-04
10–14	5.97E-04***	−1.33E-03***	8.07E-05	−9.32E-05
15–19	8.75E-04***	−1.68E-03***	1.15E-04	−1.21E-04
20–24	1.18E-03***	−2.08E-03***	1.83E-04*	−1.67E-04
25–29	1.69E-03***	−2.66E-03***	2.45E-04*	−1.57E-04
30–34	2.59E-03***	−3.55E-03***	2.73E-04	−3.45E-05
35–39	3.89E-03***	−4.59E-03***	4.02E-04	8.31E-05
40–44	5.50E-03***	−5.49E-03***	8.70E-04*	−3.35E-05
45–49	7.33E-03***	−5.96E-03***	1.89E-03***	−5.94E-04
50–54	9.96E-03***	−6.45E-03***	3.25E-03***	−1.31E-03
55–59	1.46E-02***	−8.12E-03***	4.26E-03***	−1.38E-03
60–64	2.23E-02***	−1.22E-02***	4.24E-03***	−1.64E-05
65–69	3.32E-02***	−1.94E-02***	3.33E-03*	2.37E-03
70–74	4.56E-02***	−3.04E-02***	2.94E-03	3.49E-03
75–79	5.74E-02***	−4.53E-02***	4.58E-03	9.11E-04
80–84	6.52E-02***	−6.25E-02***	9.31E-03	−7.16E-03
85–89	6.13E-02***	−7.54E-02***	1.70E-02*	−2.09E-02*
90–94	3.78E-02**	−7.64E-02***	2.75E-02**	−4.02E-02**

The number represents the coefficient, and the symbol in the upper right corner represents the significance. Table 5 shares the same format.

One asterisk (*) indicates significance at the 5% level, i.e., *p* < 0.05; two asterisks (**) indicate significance at the 1% level, i.e., *p* < 0.01; three asterisks (***) indicate significance at the 0.1% level, i.e., *p* < 0.001.

The regression coefficients for the gender variable are significant for all age groups except the 1–4-year-old age group. For neonates (0 years old), the gender variable is significant at the 0.1 level. For the 90–94 age group, the significance level for the gender variable is 0.05. For all the other age groups, the p values of the t tests for the gender variable are less than 0.01. This finding aligns with previous research confirming that male mortality probabilities are generally greater than female mortality probabilities. Our study further revealed that in each age group, under other equal conditions, the male mortality probabilities were consistently greater than the female mortality probabilities. This indicates that gender-specific mortality patterns in China have transitioned into a "modern pattern"^33^.

The urban‒rural variable has a negative influence on mortality probabilities across all age groups, and its impact is significant at the 0.001 level for all 20 age groups. This implies that, under other equal conditions, urban mortality probabilities are generally lower than rural mortality probabilities. Unlike urban areas, rural regions face challenges with limited medical facilities, scarce healthcare resources, and poor health conditions, which can impact medical accessibility. Rural areas are more vulnerable to infectious diseases due to poor infrastructure. Urban areas, with better education and health awareness, exhibit improved lifestyles and overall enhanced health. Economic advantages in urban regions enable access to advanced medical care. The hospital beds variable significantly positively impacts the mortality probability for individuals aged 20–29, 40–69, and 85–94 years. This effect may be linked to variations in healthcare facility quality. Simply having more medical beds does not guarantee lower mortality if healthcare quality is inadequate. For the fatality rate, a significant impact is noted in the 85–94 age group, and it is important to highlight that this effect is negative.

### Analysis of the effects of other indicators by age group

In Table 5, further regression analysis indicates that the variables health technicians and insured employees lack significance across all age groups, leading to their exclusion from further examination. Consequently, our focus narrows to 12 variables—4 global and 8 local. We will proceed to assess the impact of these eight remaining factors in age-specific groups, ranking them based on the magnitude of influence (sorted by the absolute values of coefficients). The results will be presented in three stages: low age group (0–29 years), mid age group (30–64 years), and high age group (65–94 years). We will discuss the noteworthy socioeconomic factors influencing each of these stages.

**Table 5:** Coefficient values and significance of t Tests for regression coefficients of other indicators in various age groups (two-tailed).

Age groups	β_2_(*t*)	β_3_(*t*)	β_5_(*t*)	β_6_(*t*)	β_7_(*t*)	β_9_(*t*)	β_11_(*t*)	β_12_(*t*)	β_13_(*t*)	β_14_(*t*)
0	1.02E-03**	2.36E-04	−5.87E-04*	−1.60E-04	−1.06E-03**	−5.67E-05	−8.70E-04*	−3.21E-05	−1.10E-03***	−1.14E-04
1–4	5.85E-04***	2.11E-04	−2.16E-04*	−1.70E-04	−5.88E-04***	−3.64E-05	−4.89E-04*	2.98E-05	−7.31E-04***	−5.90E-05
5–9	3.35E-04**	1.59E-04	−7.16E-05	−3.58E-04*	−1.39E-04	3.44E-05	−2.86E-04	1.50E-05	−5.22E-04***	−2.65E-05
10–14	2.30E-04	1.07E-04	−4.34E-05	−5.40E-04**	1.47E-04	8.90E-05	−1.92E-04	−5.52E-06	−4.32E-04***	−9.24E-06
15–19	2.27E-04	7.76E-05	−3.13E-05	−5.65E-04**	1.60E-04	7.48E-05	−1.43E-04	2.24E-05	−4.17E-04**	8.73E-07
20–24	2.77E-04	8.25E-05	1.73E-07	−5.13E-04*	−1.90E-06	3.43E-05	−1.29E-04	4.26E-05	−4.59E-04***	1.39E-05
25–29	3.24E-04	1.29E-04	6.00E-05	−5.61E-04*	−1.59E-04	4.90E-05	−1.61E-04	−4.64E-05	−5.47E-04***	4.05E-05
30–34	3.25E-04	2.29E-04	1.63E-04	−8.65E-04	−1.44E-04	1.85E-04	−2.44E-04	−3.30E-04	−6.77E-04*	9.26E-05
35–39	3.43E-04	4.39E-04	3.96E-04	−1.35E-03	8.07E-05	3.38E-04	−3.32E-04	−7.09E-04	−8.97E-04*	1.92E-04
40–44	5.14E-04	8.47E-04	8.88E-04	−1.77E-03	4.63E-04	2.91E-04	−3.45E-04	−9.66E-04	−1.30E-03*	3.68E-04
45–49	9.68E-04	1.53E-03	1.75E-03*	−1.92E-03	9.37E-04	−1.59E-04	−2.16E-04	−8.98E-04	−1.95E-03*	6.47E-04
50–54	1.72E-03	2.25E-03*	2.53E-03*	−1.89E-03	1.16E-03	−8.58E-04	−2.44E-04	−6.68E-04	−2.71E-03**	1.00E-03*
55–59	2.67E-03	2.46E-03	2.22E-03	−2.10E-03	4.80E-04	−1.29E-03	−1.09E-03	−8.11E-04	−3.19E-03*	1.36E-03*
60–64	3.71E-03	1.63E-03	−1.66E-04	−2.99E-03	−1.72E-03	−9.48E-04	−3.41E-03	−1.87E-03	−3.01E-03	1.64E-03*
65–69	4.92E-03	−2.24E-04	-4.20E-03	−4.87E-03	−5.23E-03	5.23E-04	−7.47E-03	−4.25E-03	−2.21E-03	1.77E-03
70–74	6.68E-03	−2.17E-03	−7.21E-03*	−7.87E-03	−8.52E-03	3.18E-03	−1.29E-02	−8.11E-03	−1.50E-03	1.70E-03
75–79	9.37E-03	−3.22E-03	−6.33E-03	−1.21E-02	−9.94E-03	7.05E-03	−1.92E-02	−1.36E-02	−1.61E-03	1.40E-03
80–84	1.33E-02	−3.09E-03	1.38E-04	−1.64E-02	−8.10E-03	1.14E-02	−2.58E-02	−2.04E-02	−2.87E-03	1.21E-03
85–89	1.88E-02	−3.33E-03	1.10E-02	−1.62E-02	−2.20E-03	1.37E-02	−3.17E-02	−2.71E-02	−4.45E-03	2.42E-03
90–94	2.59E-02	−5.72E-03	2.48E-02	−6.66E-03	8.47E-03	1.10E-02	−3.59E-02	−3.20E-02	−5.37E-03	6.48E-03

One asterisk (*) indicates significance at the 5% level, i.e., *p* < 0.05; two asterisks (**) indicate significance at the 1% level, i.e., *p* < 0.01; three asterisks (***) indicate significance at the 0.1% level, i.e., *p* < 0.001.

#### Analysis of the impact of socioeconomic factors on mortality patterns—low age group (0–29 years)

**Figure 4:**
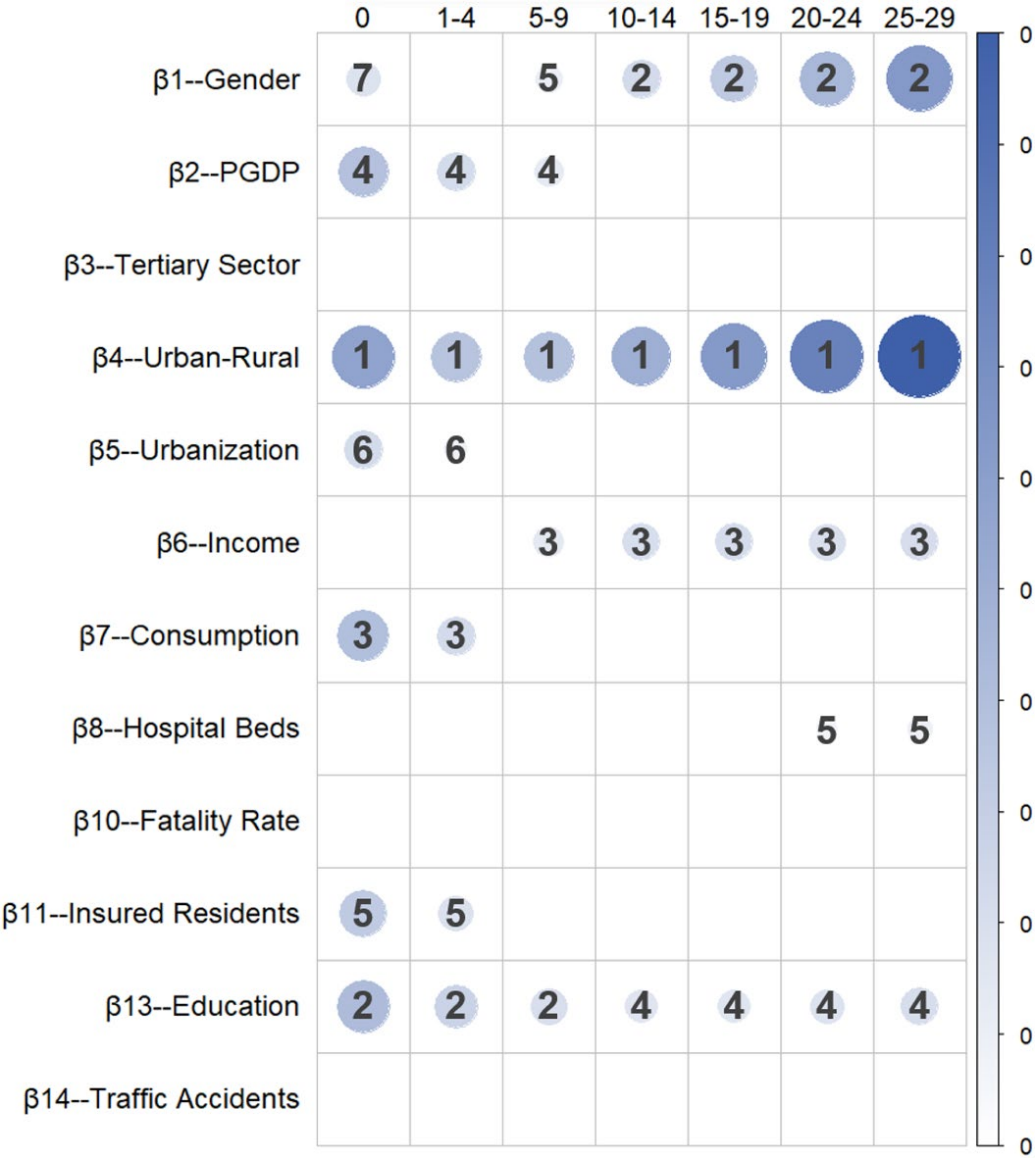
Marginal impact ranking of factors in the low age group. Note: The size and color intensity of the circles represent the magnitude of the absolute values, where larger circles with darker colors indicate larger absolute values. (The same applies throughout).

Table 6 and Fig. 4 present the marginal effects and ranking of various factors in the mortality patterns for the low age group.

**Table 6:** Absolute values, significance, and ranking of the regression coefficients for the low age group.

Ranking	0	1–4	5–9	10–14	15–19	20–24	25–29
1	β_4_-UR 1.54E-03 4.45E-11***	β_4_-UR 9.83E-04 5.74E-20***	β_4_-UR 1.02E-03 1.76E-26***	β_4_-UR 1.33E-03 6.01E-26***	β_4_-UR 1.68E-03 3.26E-31***	β_4_-UR 2.08E-03 1.79E-39***	β_4_-UR 2.66E-03 2.71E-40***
2	β_13_-EDU 1.10E-03 3.92E-05***	β_13_-EDU 7.31E-04 1.22E-09***	β_13_-EDU 5.22E-04 1.23E-07***	β_1_-GD 5.97E-04 8.83E-09***	β_1_-GD 8.75E-04 9.46E-14***	β_1_-GD 1.18E-03 6.31E-21***	β_1_-GD 1.69E-03 9.03E-25***
3	β_7_-PCC 1.06E-03 3.32E-03**	β_7_-PCC 5.88E-04 2.70E-04***	β_6_-PCI 3.58E-04 2.48E-02*	β_6_-PCI 5.40E-04 9.31E-03**	β_6_-PCI 5.65E-04 8.60E-03**	β_6_-PCI 5.13E-04 1.24E-02*	β_6_-PCI 5.61E-04 2.97E-02*
4	β_2_-GDP 1.02E-03 3.10E-03**	β_2_-GDP 5.85E-04 1.35E-04***	β_2_-GDP 3.35E-04 9.28E-03**	β_13_-EDU 4.32E-04 5.83E-04***	β_13_-EDU 4.17E-04 1.58E-03**	β_13_-EDU 4.59E-04 3.80E-04***	β_13_-EDU 5.47E-04 7.19E-04***
5	β_11_-IR 8.70E-04 4.00E-02*	β_11_-IR 4.89E-04 1.16E-02*	β_1_-GD 2.77E-04 1.89E-04***			β_8_-HB 1.83E-04 2.16E-02*	β_8_-HB 2.45E-04 1.47E-02*
6	β_5_-URR 5.87E-04 1.86E-02*	β_5_-URR 2.16E-04 4.16E-02*					
7	β_1_-GD 4.68E-04 2.80E-02*						

In each cell, the first row represents the indicator name, the second row denotes the absolute coefficient values, and the third row indicates the p value along with the significance symbols. Tables 7 and 8 share the same format.

One asterisk (*) indicates significance at the 5% level, i.e., *p* < 0.05; two asterisks (**) indicate significance at the 1% level, i.e., *p* < 0.01; three asterisks (***) indicate significance at the 0.1% level, i.e., *p* < 0.001.

From Table 6 and Fig. 4, it is clear from the results that the urban‒rural factor ranks first in terms of its impact on the seven population groups within the low age range. After urban‒rural, the variables education and gender follow, with gender having a more significant impact on newborns (0 years) and young children (5–9 years), whereas gender has no significant influence on the 1- to 4-year-old age group. However, the influence of gender begins to increase in the subsequent 10–29-year age group, ranking second in terms of its effect on mortality probabilities. Newborns are affected by seven significant factors, while the 10–14-year-old and 15–19-year-old groups are influenced by only four factors.

Education significantly influences the 0–29 years age group, with p values mostly below 0.01 (except for the 15–19 age group). Higher education has a notable negative impact on mortality, particularly in the 0–9 age range, ranking second in influencing infant and toddler mortality. Overall, women with higher education levels exhibit increased self-care and prevention behaviors, contributing to reduced infant mortality probabilities^0^. In the 10–29 age group, education ranks fourth in terms of the degree of impact. Individuals with higher education levels tend to adopt healthier lifestyles and possess more health awareness.

Income reflects people's living standards and purchasing power. The mortality probability for the age group of 5–29 years is significantly influenced by income, ranking it as the third most influential factor. The data show that income has the most significant impact on the 15- to 19-year-old population, reducing mortality probability by 5.65E-4.

The proportion of urban and rural residents covered by basic medical insurance has a significant impact on the 0–4-year-old age group, with the most pronounced effect occurring for the 1–4-year-old age group, where the influence value peaks at -4.89E-4.

The urbanization rate variable significantly affects the mortality probabilities of infants and young children aged 0–4 years, ranking as the sixth most influential factor. For each percentage point increase in the urbanization rate, the mortality probability for newborns decreases by 5.87E-4, and for 1–4-year-olds, the mortality probability decreases by 2.16E-4. Urban areas have superior medical facilities, more healthcare institutions, and more advanced medical services, ensuring that residents access higher-quality medical resources^5^.

The variable per capita GDP significantly affects infants and young children aged 0–9 years, consistently ranking fourth. However, the findings of this study reveal a positive influence of per capita GDP on infant mortality probabilities, which warrants further discussion.

#### *Analysis of the impact *of* socioeconomic factors on mortality patterns—mid age group (30–64 years)*

**Figure 5:**
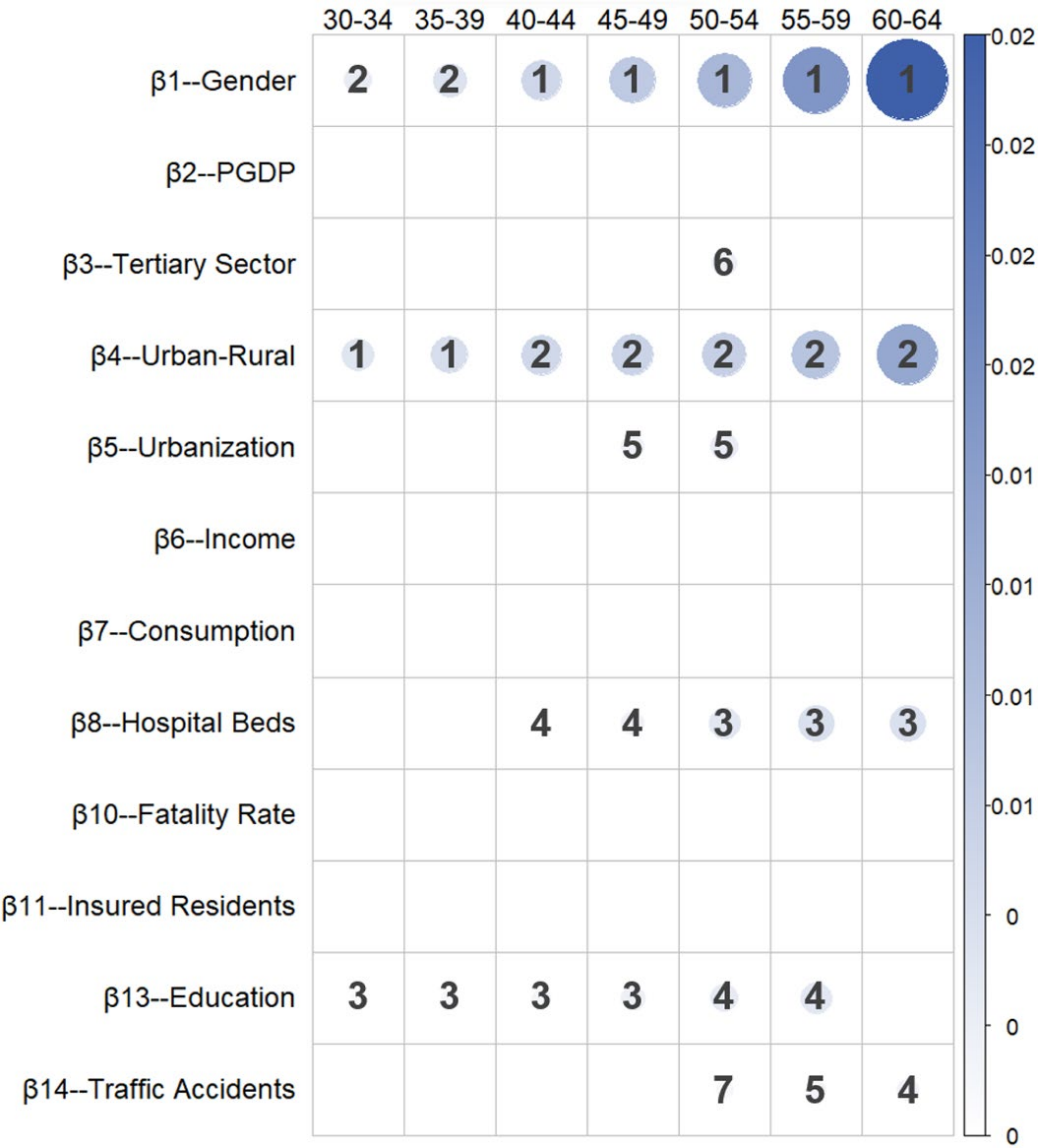
Marginal impact ranking of factors in the mid age group.

Table 7 and Fig. 5 present the marginal effects and ranking of various factors on the mortality patterns for the mid age group.

**Table 7:** Absolute values, significance, and ranking of the regression coefficients for the mid age group.

Ranking	30–34	35–39	40–44	45–49	50–54	55–59	60–64
1	β_4_-UR 3.55E-03 6.09E-32***	β_4_-UR 4.59E-03 3.94E-24***	β_1_-GD 5.50E-03 7.18E-19***	β_1_-GD 7.33E-03 1.23E-19***	β_1_-GD 9.96E-03 3.22E-22***	β_1_-GD 1.46E-02 1.57E-26***	β_1_-GD 2.23E-02 2.58E-31***
2	β_1_-GD 2.59E-03 3.28E-22***	β_1_-GD 3.89E-03 1.24E-19***	β_4_-UR 5.49E-03 7.86E-19***	β_4_-UR 5.96E-03 6.99E-15***	β_4_-UR 6.45E-03 2.03E-12***	β_4_-UR 8.12E-03 2.17E-12***	β_4_-UR 1.22E-02 9.69E-15***
3	β_13_-EDU 6.77E-04 1.26E-02*	β_13_-EDU 8.97E-04 4.56E-02*	β_13_-EDU 1.30E-03 4.71E-02*	β_13_-EDU 1.95E-03 2.08E-02*	β_8_-HB 3.25E-03 1.45E-06***	β_8_-HB 4.26E-03 5.87E-07***	β_8_-HB 4.24E-03 1.22E-04***
4			β_8_-HB 8.70E-04 3.35E-02*	β_8_-HB 1.89E-03 4.15E-04***	β_13_-EDU 2.71E-03 9.02E-03**	β_13_-EDU 3.19E-03 1.45E-02*	β_14_-TA 1.64E-03 4.93E-02*
5				β_5_-URR 1.75E-03 2.94E-02*	β_5_-URR 2.53E-03 1.05E-02*	β_14_-TA 1.36E-03 3.13E-02*	
6					β_3_-TS 2.25E-03 4.73E-02*		
7					β_14_-TA 1.00E-03 4.51E-02*		

One asterisk (*) indicates significance at the 5% level, i.e., *p* < 0.05; two asterisks (**) indicate significance at the 1% level, i.e., *p* < 0.01; three asterisks (***) indicate significance at the 0.1% level, i.e., *p* < 0.001.

Among the socioeconomic factors significantly affecting the mortality probabilities of mid age group, the factor gender gradually surpasses the urban‒rural factor to become the primary factor. In addition, the most pronounced effect is observed for the education indicator, which ranks third in terms of its influence on the 30–49-year-old population and then falls to fifth place among the 50–59-year-old population, with no significant impact on the 60–64-year-old group. Educational attainment is often associated with occupation and economic status. Higher educational levels are typically accompanied by better employment opportunities and higher income levels, thus reducing health risks.

The urbanization rate significantly impacts the mortality probabilities of mid age group, particularly individuals aged 45–54, ranking fifth in terms of its influence. The effect is positive, implying that urban living might be associated with greater social and economic pressures, which can negatively impact the health of middle-aged individuals. Similarly, the proportion of the tertiary sector significantly affects the mortality probability of the 50- to 54-year-old group. With increasing age, traffic accidents have a progressively greater impact on the mortality probability, with coefficient values showing an upward trend from 9.26E-5 to 1.64E-3 and significant effects among the 50- to 64-year-old population.

#### *Analysis of the impact of socioeconomic factors on mortality patterns—high age *group* (65–94 years)*

**Figure 6:**
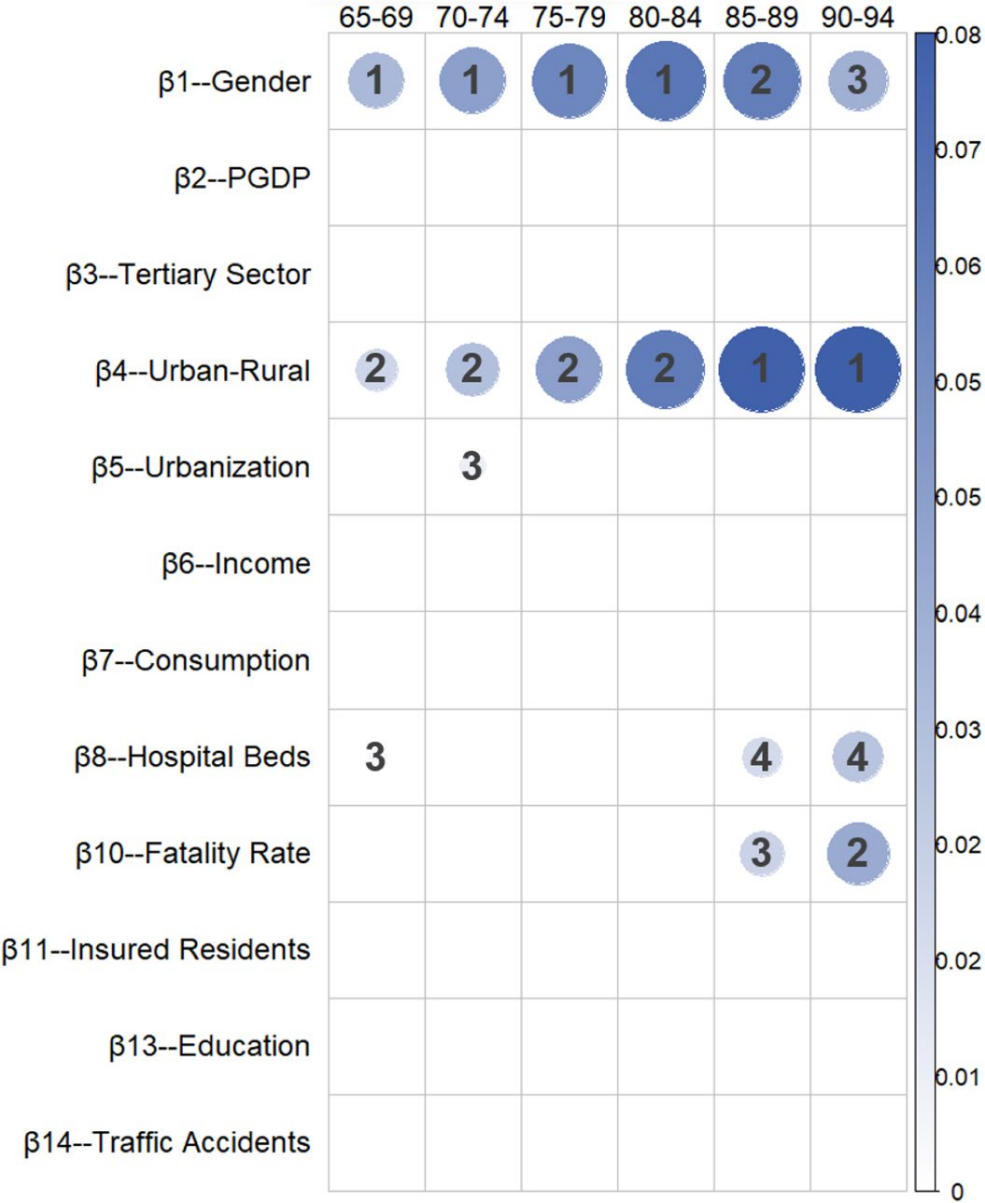
Marginal impact ranking of factors in the high age group.

Table 8 and Fig. 6 present the marginal effects and ranking of various factors in the mortality patterns for the high age group.

**Table 8:** Absolute values, significance, and ranking of the regression coefficients for the high age group.

Ranking	65–69	70–74	75–79	80–84	85–89	90–94
1	β_1_-GD 3.32E-02 4.93E-33***	β_1_-GD 4.56E-02 1.94E-29***	β_1_-GD 5.74E-02 9.64E-23***	β_1_-GD 6.52E-02 1.23E-15***	β_4_-UR 7.54E-02 3.27E-12***	β_4_-UR 7.64E-02 5.85E-09***
2	β_4_-UR 1.94E-02 2.33E-17***	β_4_-UR 3.04E-02 6.44E-18***	β_4_-UR 4.53E-02 9.57E-17***	β_4_-UR 6.25E-02 9.01E-15***	β_1_-GD 6.13E-02 4.43E-09***	β_10_-FR 4.02E-02 1.05E-03**
3	β_8_-HB 3.33E-03 2.99E-02*	β_5_-URR 7.21E-03 4.34E-02*			β_10_-FR 2.09E-02 2.94E-02*	β_1_-GD 3.78E-02 2.25E-03**
4					β_8_-HB 1.70E-02 2.59E-02*	β_8_-HB 2.75E-02 4.55E-03**

One asterisk (*) indicates significance at the 5% level, i.e., *p* < 0.05; two asterisks (**) indicate significance at the 1% level, i.e., *p* < 0.01; three asterisks (***) indicate significance at the 0.1% level, i.e., *p* < 0.001.

In the elderly population, the main influencing factors are the four global variables analyzed earlier. In the 65–84-year-old group, the primary influencing factor is the gender, followed by the urban‒rural factor. However, among the 85–94-year-old population, the influence of gender is lower than that of urban‒rural.

The urbanization rate has a more significant impact on the mortality probability of individuals aged 70–74 years. Education has no significant effect on the mortality probability of the high age group compared with the low and mid age groups. Some research has suggested that education might not be a strong indicator of the socioeconomic status of China's elderly population^15^.

For the elderly population, the chosen variables inadequately explain the mortality probabilities, suggesting that additional factors play a crucial role in this age group. Age-related factors are prominent as the body undergoes natural aging, increasing the likelihood of mortality. Socioeconomic factors are not the primary determinants for this elderly segment.

In the high age group, some factors exhibit large coefficient values but remain nonsignificant. For example, both insured residents and insured employees have substantial negative effects on the mortality probability, and their impact becomes more pronounced with increasing age. Furthermore, income and consumption also have a certain effect on reducing the mortality probability of the elderly, but these factors were not significant.

## Conclusion

Leveraging data from China’s 7th Population Census, our research delved into the socioeconomic impact factors and mechanisms of mortality patterns, accounting for interprovincial disparities, urban‒rural divides, and gender differences. This study also scrutinized their marginal effects and variations across age groups. The key findings include:

1. Regional mortality patterns in China show significant gender and urban‒rural disparities across most age groups. In the same age group, the mortality probabilities are generally greater for males than for females, and they generally are greater in rural areas than in urban areas. The factor urban‒rural dominates the mortality probabilities of individuals aged 39 and younger, while the factor gender becomes most significant in the 40–84 age group.
2. Socioeconomic factors, apart from gender and urban‒rural distinctions, on mortality patterns typically become evident after the age of 45, with less noticeable variations in their impact on early-life mortality patterns.
3. Factors such as PGDP, tertiary sector, urban‒rural, urbanization, income, consumption, hospital beds, fatality rate, insured residents, education, and traffic accidents have different influence directions on mortality patterns across age groups, and the marginal effects of each factor are different. For example, in the 0–29 age group, education exerts a significant negative effect on mortality patterns, and the marginal effect is larger than that of most other factors. The education index still has a negative effect on the mortality probabilities of the population aged 30–59, and there is no significant effect on the high age group. The rate of urbanization exerts a significant positive influence on the mortality probability of the 45–54 age group. With increasing age, the impact of traffic accidents on the mortality probability gradually increases. The mortality pattern of the elderly group is mainly affected by the factors hospital beds and fatality rate, but other socioeconomic variables have no significant affected.
4. The socioeconomic indicator mechanism displays an age structure effect, with lower explanatory power for mortality patterns in older age groups. Further analysis in older age groups should consider additional explanatory variables.

These conclusions contribute significantly to our understanding of the impact of socioeconomic factors on mortality patterns according to demographic data. They underscore the complexity of socioeconomic factors' influence on mortality patterns and deepen our comprehension of age-specific differences in mortality patterns.

This paper has limitations, primarily focusing on the influence of socioeconomic factors while overlooking biological and environmental factors. This limitation results in a relatively low explanatory power of the model, especially for the mortality probabilities of elderly people. Future research will explore the impact of geographical and environmental resource factors on mortality patterns and their mechanisms.

### Supplementary Information

Supplementary Information.

## Data Availability

The data that support the findings of this study are available upon request from the corresponding author.
