# Supplementary material for: Research on social and economic factors influencing regional mortality patterns in China
Source: Sci Rep. 2024 May 9;14:10614. doi: 10.1038/s41598-024-61262-5 (PMC11078968; doi:10.1038/s41598-024-61262-5)
Supplement: Supplementary file 1 — Supplementary Information. [file 41598_2024_61262_MOESM1_ESM.pdf]

Research on social and economic factors influencing regional mortality patterns in China

Tiantian Li[1] . Shuyin Zhang[1] . Handong Li[1,*]

[1]Beijing Normal University (School of Systems Science), Beijing, China.

[*]Corresponding author. Email: lhd@bnu.edu.cn

Journal

Scientific Reports

Supplementary Information I

Online Resource 1

We utilized data from the National Bureau of Statistics (https://www.stats.gov.cn) website, which is freely available on their website. The data retrieval date was in May 2022.

We utilized data sourced from the National Bureau of Statistics 2020 *China Population Census Yearbook* (http://www.stats.gov.cn/sj/pcsj/rkpc/7rp/zk/indexch.htm), the 2021 *China Health Statistics Yearbook* (http://www.nhc.gov.cn/mohwsbwstjxxzx/tjzxtjsj/tjsj_list.shtml), and annual provincial-level data from the National Data Website (https://data.stats.gov.cn/easyquery.htm?cn=E0103). This data is freely available on their respective websites. The data retrieval date was in May 2023.

Supplementary Information II

In this section, we conducted multiple regression analyses on the factors influencing the mortality probabilities in various age groups and compared some of the results.

Firstly, in Appendix Table 1, we compared the coefficients of determination (R-squared) between Functional Data Analysis (FDA) and Multiple Linear Regression (MLR). The first row presents the results of the Functional Data Analysis (FDA), while the second row displays the results of Multiple Linear Regression (MLR).

It can be observed that in the age-specific multiple regression analysis, the goodness of fit for the lower age group (0-24) and the elderly group (65-94) is lower compared to the Functional Data Analysis method. In the study of factors influencing death levels, particular attention needs to be given to the data of infants and the elderly. Therefore, considering the goodness of fit in these age groups, the Functional Data Analysis method demonstrates superiority.

Appendix Table 1 Comparison of R^2 from FDA and MLR

Age groups	0	1-4	5-9	10-14	15-19	20-24	25-29	30-34	35-39	40-44
**FDA**	0.6754	0.6813	0.7567	0.805	0.8097	0.7956	0.7888	0.7910	0.7741	0.7420
**MLR**	0.6214	0.6424	0.7266	0.705	0.7919	0.7763	0.7863	0.7686	0.7596	0.7675

Age groups	45-49	50-54	55-59	60-64	65-69	70-74	75-79	80-84	85-89	90-94
**FDA**	0.6744	0.6245	0.6648	0.7758	0.8205	0.7848	0.7100	0.6058	0.4993	0.4334

**MLR**	0.7864	0.7818	0.8037	0.8236	0.8058	0.7505	0.6982	0.5852	0.4957	0.4571

Furthermore, Functional Data Analysis focuses on the influencing factors of the overall mortality pattern, while traditional multivariate analysis mainly concentrates on the factors of a specific age group. As a result, traditional multivariate analysis may not accurately identify significant factors affecting the overall death pattern (such as gender and urban-rural factors), whereas Functional Data Analysis, with its unique functional tests, provides a more comprehensive understanding of significant factors affecting the entire pattern.

Lastly, when applying the regression results obtained from Functional Data Analysis from a holistic perspective to specific age groups, some differences exist compared to the results obtained using traditional multiple regression methods. Appendix Tables 2 and 3 present the results of multiple regression analyses for the 0-year-old and 45-49-year-old populations, respectively.

Appendix Table 2 Multiple regression results -- 0 years old

	**Estimate**	**Std. Error**	**t value**	**Pr(>|t|)**
(Intercept)	1.869e-02	2.418e-03	7.730	5.7e-12 ***
Gender	3.975e-04	2.069e-04	1.921	0.057286 .
PGDP	1.626e-08	1.020e-08	1.593	0.114001
Tertiary Sector	1.399e-05	3.812e-05	0.367	0.714353
Urban-Rural	-9.236e-04	7.048e-04	-1.310	0.192807
Urbanization	-5.702e-05	1.827e-05	-3.121	0.002306 **
Income	6.338e-08	4.867e-08	1.302	0.195513
Consumption	-1.789e-07	8.364e-08	-2.139	0.034691 *
Hospital Beds	-6.274e-06	1.860e-05	-0.337	0.736586
Health Technicians	6.879e-07	1.669e-05	0.041	0.967201
Fatality Rate	1.267e-04	5.251e-04	0.241	0.809774
Insured Residents	-4.172e-05	2.203e-05	-1.894	0.060895 .
Insured Employees	-2.512e-05	4.889e-05	-0.514	0.608441
Education	-9.027e-04	2.597e-04	-3.475	0.000733 ***
Traffic Accidents	-2.433e-08	2.051e-08	-1.186	0.238033

Note: Signif. codes: 0 ‘***’ 0.001 ‘**’ 0.01 ‘*’ 0.05 ‘.’ 0.1 ‘ ’ 1

Appendix Table 3 Multiple regression results – 45-49 years old

	**Estimate**	**Std. Error**	**t value**	**Pr(>|t|)**
(Intercept)	5.009e-03	7.158e-03	0.700	0.486
Gender	8.543e-03	6.124e-04	13.951	< 2e-16 ***
PGDP	-3.557e-08	3.020e-08	-1.178	0.241
Tertiary Sector	7.045e-06	1.129e-04	0.062	0.950
Urban-Rural	-8.603e-03	2.086e-03	-4.123	7.31e-05 ***
Urbanization	2.588e-05	5.407e-05	0.479	0.633
Income	1.619e-09	1.441e-07	0.011	0.991
Consumption	7.833e-08	2.476e-07	0.316	0.752
Hospital Beds	8.884e-05	5.507e-05	1.613	0.110
Health Technicians	3.561e-05	4.941e-05	0.721	0.473
Fatality Rate	9.778e-04	1.554e-03	0.629	0.531
Insured Residents	3.822e-05	6.521e-05	0.586	0.559

Insured Employees	-3.818e-05	1.447e-04	-0.264	0.792
Education	-6.655e-04	7.689e-04	-0.866	0.389
Traffic Accidents	6.098e-08	6.071e-08	1.004	0.317

Note: Signif. codes: 0 '***' 0.001 '**' 0.01 '*' 0.05 '.' 0.1 ' ' 1

In the multiple regression analysis of the mortality level for the 0-year-old population, it was observed that gender and urban-rural factors were not significant at a significance level of 0.05. However, through functional regression analysis, it was revealed that these two factors actually have a global impact on the overall mortality level. Additionally, both PGDP and Insured Residents did not exhibit significance in the multiple regression analysis. It is noteworthy that the marginal effects of these influencing factors differ between functional regression analysis and multiple regression. For the middle-aged population, multiple regression analysis failed to reveal the impact of education level, urbanization rate, and the number of hospital beds on the death patterns.

Multiple regression, limited in considering overall changes, is susceptible to noise interference, making it challenging to identify significant factors. In contrast, functional regression circumvents this issue. Taking education as an example, in functional regression analysis, it exhibits a significant influence on the age group of 0-59, with varying marginal effects. However, when separately conducting multiple regression analyses for these age groups, it was found that education did not significantly affect individuals aged 15-19 and those aged 25 and above. This discrepancy is evidently contrary to common perceptions. Previous studies have highlighted the significant impact of education on mortality rates in China, a conclusion not consistently derived from multiple regression analyses conducted across various age groups.
